# Dichlorido(2-chloro-9-mesityl-1,10-phenanthroline-κ^2^ *N*,*N*′)cobalt(II) dichloro­methane hemisolvate

**DOI:** 10.1107/S1600536808007320

**Published:** 2008-04-04

**Authors:** Yue Yang, Peiju Yang, Cui Zhang, Biao Wu

**Affiliations:** a State Key Laboratory for Oxo Synthesis & Selective Oxidation, Lanzhou Institute of Chemical Physics, CAS, Lanzhou 730000, People’s Republic of China; b Graduate University of the Chinese Academy of Sciences, Beijing 100049, People’s Republic of China

## Abstract

The title compound, [CoCl_2_(C_21_H_17_ClN_2_)]·0.5CH_2_Cl_2_, crystallizes from dichloro­methane as a 2:1 solvate [CoCl_2_L]_2_·CH_2_Cl_2_ (*L* is 2-chloro-9-mesityl-1,10-phenanthroline). There are two independent CoCl_2_ *L* mol­ecules in the asymmetric unit and both mol­ecules have similar conformations. They are connected by a weak C—H⋯π inter­action involving the mesityl ring. The cobalt center is four-coordinated by the two N-atom donors of the bidentate ligand and two chloride ions in a distorted tetra­hedral geometry. The packing of the mol­ecules is stabilized by weak slipped π–π stacking inter­actions between symmetry-related phenanthroline groups.

## Related literature

For related literature, see: Britovsek *et al.* (1998 ▶); Garas & Vagg (2000 ▶); Gibson & Spitzmesser (2003 ▶); Sauvage (1990 ▶); Small & Brookhart (1998 ▶).
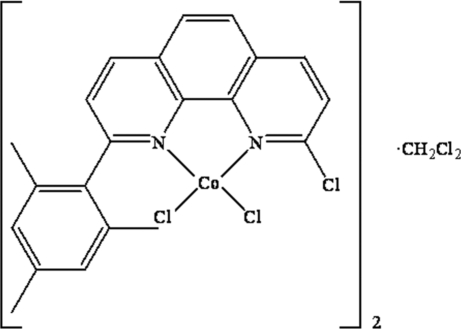


## Experimental

### 

#### Crystal data

[CoCl_2_(C_21_H_17_ClN_2_)]·0.5CH_2_Cl_2_
*M* *_r_* = 505.11
Triclinic, 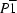

*a* = 9.8830 (3) Å
*b* = 15.3591 (5) Å
*c* = 15.6544 (5) Å
α = 79.964 (1)°
β = 78.094 (1)°
γ = 74.515 (1)°
*V* = 2222.75 (12) Å^3^
*Z* = 4
Mo *K*α radiation
μ = 1.26 mm^−1^
*T* = 293 (2) K
0.45 × 0.36 × 0.25 mm

#### Data collection

Bruker APEXII diffractometer
Absorption correction: multi-scan (*SADABS*; Bruker, 1999 ▶) *T*_min_ = 0.688, *T*_max_ = 1.000 (expected range = 0.501–0.729)
13275 measured reflections
9215 independent reflections
5134 reflections with *I* > 2σ(*I*)
*R*_int_ = 0.025

#### Refinement

*R*[*F* ^2^ > 2σ(*F* ^2^)] = 0.056
*wR*(*F* ^2^) = 0.159
*S* = 1.02
9215 reflections
520 parameters
H-atom parameters constrained
Δρ_max_ = 0.44 e Å^−3^
Δρ_min_ = −0.69 e Å^−3^

### 

Data collection: *APEX2* (Bruker, 2004 ▶); cell refinement: *SAINT* (Bruker, 2004 ▶); data reduction: *SAINT*; program(s) used to solve structure: *SHELXS97* (Sheldrick, 2008 ▶); program(s) used to refine structure: *SHELXL97* (Sheldrick, 2008 ▶); molecular graphics: *ORTEP-3 for Windows* (Farrugia, 1997 ▶); software used to prepare material for publication: *SHELXL97*.

## Supplementary Material

Crystal structure: contains datablocks I, global. DOI: 10.1107/S1600536808007320/dn2321sup1.cif

Structure factors: contains datablocks I. DOI: 10.1107/S1600536808007320/dn2321Isup2.hkl

Additional supplementary materials:  crystallographic information; 3D view; checkCIF report

## Figures and Tables

**Table 1:** Hydrogen-bond geometry (Å, °)

*D*—H⋯*A*	*D*—H	H⋯*A*	*D*⋯*A*	*D*—H⋯*A*
C9—H9⋯*Cg*1^i^	0.93	2.59	3.470 (5)	157

Symmetry code: (i) 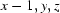. *Cg*1 is the centroid of the mesityl ring.

**Table 2:** Possible π–π stacking interactions (Å, °)

	Centroid–centroid	α	*CgI*-perp	*CgJ*-perp	slippage
*Cg*2 – *Cg*2^i^	3.877 (3)	0.0	3.712	3.712	1.12
*Cg*2 – *Cg*3^i^	3.923 (3)	0.49	3.712	3.701	1.23
*Cg*4 – *Cg*4^ii^	3.992 (3)	0.02	3.490	3.490	1.94

Symmetry codes: (i) 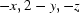; (ii) 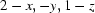. *CgI* –*CgJ* = distance between ring centroids; α = dihedral angle between planes *I* and *J*; *CgI*–perp = perpendicular distance of *Cg*(*I*) from ring *J*; *CgJ*-perp = perpendicular distance of *Cg*(*J*) from ring *I*; slippage = distance between *Cg*(*I*) and perpendicular projection of *Cg*(*J*) on ring *I*. *Cg*2 is the centroid of atoms N1,C1–C4,C12; *Cg*3 is the centroid of atoms C4–C7,C11,C12; *Cg*4 is the centroid of atoms N3,C22–C25,C33.

**Fig. 1.**
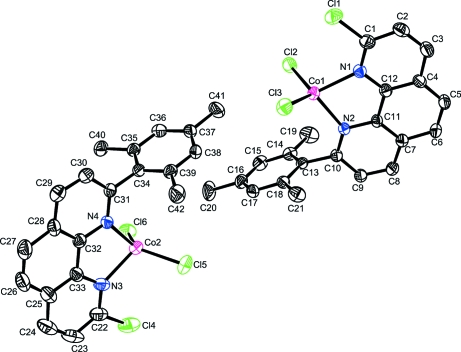
The molecular structure of the title compound, with the atom-numbering scheme. Displacement ellipsoids are drawn at the 30% probability level. H atoms and the solvent molecule have been omitted for clarity.

## supplementary crystallographic information

### Comment

Since Co^II^ complexes have been found to have high catalytic activities for the ethylene polymerization, much research interest has been inspired in cobalt metal catalysis systems over the past decade (Small & Brookhart, 1998; Britovsek *et al.*,1998). Research in this area frequently involves the design of new ancillary ligands to support and activate the metal center toward polymerization (Gibson & Spitzmesser, 2003). 1,10-Phenanthroline and its derivatives are well established ligands in transition metal coordination chemistry because their steric and electronic environment can be conveniently tailored by varying the substituents (Sauvage,1990). The title complex is one of cobalt^II^ dihalide complexes which we have designed and its crystal structure is presented here.

The asymmetric unit contains two independent CoCl_2_L molecules with similar conformation and a CH_2_Cl_2_ solvent molecule (Fig. 1). The two CoCl_2_L units are connected by a weak C—H···π interaction involving the mesityl ring (Table 1). The cobalt center is four-coordinated by the two nitrogen donors of the bidentate ligand and two chloride ions forming a distorted tetrahedron, with the dihedral angle of the N—Co—N and Cl—Co—Cl planes being 88.53/88.52°. The dihedral angle between the phenanthroline moiety and the attached mesityl substituent is 85.51/83.42°.

The packing of the molecules is stabilized by weak slipped π-π stacking interactions between symmetry related phenanthroline rings (Table 2).

### Experimental

The ligand 2-chloro-9-mesityl-1,10-phenanthroline was synthesized according to a modified procedure (Garas & Vagg, 2000) as a pale yellow solid in 62.0% yield. *M*.p.: 515–516 K. ESI-MS: *m/z* 333.3 [*M*+H]^+. 1^H NMR (CDCl_3_, δ/p.p.m.): 8.28 (1*H*, d, J = 8.4 Hz, H4), 8.20 (1*H*, d, J = 8.8 Hz, H7), 7.86 (1*H*, d, J = 8.8 Hz, H3), 7.80 (1*H*, d, J = 8.8 Hz, H8), 7.61 (1*H*, d, J = 8.8 Hz, H5), 7.59 (1*H*, d, J = 8.8 Hz, H6), 6.97 (2*H*, s, Ph—H), 2.35 (3*H*, s, *p*-CH3), 2.13 (6*H*, s, *o*-CH3). And the title compound was readily synthesized in excellent yield through the following method: a solution of CoCl_2_.6H_2_O (0.80 g, 0.0034 mol) and the ligand (1.12 g, 0.0034 mol) in tetrahydrofuran was stirred at room temperature for 12 h, giving a light green suspension. The precipitate was collected, washed repeatedly with diethyl ether and dried under vacuum to yield the title compound (1.48 g, 95.6%). Mp: > 573 K. Anal. Calcd for C_21_H_17_N_2_CoCl_3_ (462.66): C 54.52, H 3.70, N 6.05%; Found: C 54.46, H 3.68, N 6.10%.

### Refinement

All H atoms attached to C atoms were fixed geometrically and treated as riding with C—H = 0.96 Å (methyl), 0.97Å (methylene) or 0.93 Å (aromatic) with *U*_iso_(H) = 1.2*U*_eq_(C) or *U*_iso_(H) = 1.5*U*_eq_(methyl).

### Crystal data

**Table:**

[CoCl_2_(C_21_H_17_ClN_2_)]·0.5CH_2_Cl_2_	*Z* = 4
*M**_r_* = 505.11	*F*_000_ = 1024
Triclinic, *P*1	*D*_x_ = 1.509 Mg m^−^^3^
Hall symbol: -P 1	Mo *K*α radiation λ = 0.71073 Å
*a* = 9.8830 (3) Å	Cell parameters from 2211 reflections
*b* = 15.3591 (5) Å	θ = 2.4–20.6º
*c* = 15.6544 (5) Å	µ = 1.26 mm^−^^1^
α = 79.964 (1)º	*T* = 293 (2) K
β = 78.094 (1)º	Prism, blue
γ = 74.515 (1)º	0.45 × 0.36 × 0.25 mm
*V* = 2222.75 (12) Å^3^	

### Data collection

**Table:**

Bruker APEXII diffractometer	9215 independent reflections
Radiation source: fine-focus sealed tube	5134 reflections with *I* > 2σ(*I*)
Monochromator: graphite	*R*_int_ = 0.025
*T* = 293(2) K	θ_max_ = 26.8º
ω scans	θ_min_ = 2.1º
Absorption correction: multi-scan(SADABS; Bruker, 1999)	*h* = −12→9
*T*_min_ = 0.688, *T*_max_ = 1.000	*k* = −19→18
13275 measured reflections	*l* = −19→17

### Refinement

**Table:**

Refinement on *F*^2^	Secondary atom site location: difference Fourier map
Least-squares matrix: full	Hydrogen site location: inferred from neighbouring sites
*R*[*F*^2^ > 2σ(*F*^2^)] = 0.056	H-atom parameters constrained
*wR*(*F*^2^) = 0.159	*w* = 1/[σ^2^(*F*_o_^2^) + (0.0717*P*)^2^] where *P* = (*F*_o_^2^ + 2*F*_c_^2^)/3
*S* = 1.02	(Δ/σ)_max_ = 0.002
9215 reflections	Δρ_max_ = 0.44 e Å^−^^3^
520 parameters	Δρ_min_ = −0.69 e Å^−^^3^
Primary atom site location: structure-invariant direct methods	Extinction correction: none

### Special details

**Table:**

Geometry. All e.s.d.'s (except the e.s.d. in the dihedral angle between two l.s. planes) are estimated using the full covariance matrix. The cell e.s.d.'s are taken into account individually in the estimation of e.s.d.'s in distances, angles and torsion angles; correlations between e.s.d.'s in cell parameters are only used when they are defined by crystal symmetry. An approximate (isotropic) treatment of cell e.s.d.'s is used for estimating e.s.d.'s involving l.s. planes.
Refinement. Refinement of *F*^2^ against ALL reflections. The weighted *R*-factor *wR* and goodness of fit *S* are based on *F*^2^, conventional *R*-factors *R* are based on *F*, with *F* set to zero for negative *F*^2^. The threshold expression of *F*^2^ > σ(*F*^2^) is used only for calculating *R*-factors(gt) *etc*. and is not relevant to the choice of reflections for refinement. *R*-factors based on *F*^2^ are statistically about twice as large as those based on *F*, and *R*- factors based on ALL data will be even larger.

### Fractional atomic coordinates and isotropic or equivalent isotropic displacement parameters (Å^2^)

**Table:**

	*x*	*y*	*z*	*U*_iso_*/*U*_eq_	
Co1	0.32238 (6)	0.79829 (4)	0.05811 (4)	0.04711 (18)	
Co2	0.80414 (6)	0.23178 (4)	0.34801 (4)	0.0570 (2)	
Cl1	0.41735 (14)	0.98271 (9)	−0.08111 (9)	0.0757 (4)	
Cl2	0.31739 (13)	0.86836 (8)	0.17136 (8)	0.0665 (3)	
Cl3	0.52745 (12)	0.71461 (9)	−0.00237 (9)	0.0693 (4)	
Cl4	0.7295 (2)	0.02459 (11)	0.43135 (13)	0.1119 (6)	
Cl5	0.66100 (16)	0.23526 (12)	0.25601 (12)	0.0995 (5)	
Cl6	0.72549 (15)	0.27227 (9)	0.48148 (9)	0.0774 (4)	
Cl7	0.7761 (2)	−0.26354 (17)	0.52141 (14)	0.1424 (8)	
Cl8	0.6046 (3)	−0.16227 (19)	0.3978 (2)	0.2024 (14)	
N1	0.2218 (3)	0.8907 (2)	−0.0376 (2)	0.0470 (8)	
N2	0.1439 (3)	0.7478 (2)	0.0720 (2)	0.0404 (8)	
N3	0.9359 (4)	0.1022 (2)	0.3618 (2)	0.0610 (10)	
N4	0.9946 (3)	0.2638 (2)	0.2955 (2)	0.0459 (8)	
C1	0.2594 (5)	0.9605 (3)	−0.0895 (3)	0.0565 (12)	
C2	0.1767 (5)	1.0170 (3)	−0.1498 (3)	0.0645 (13)	
H2	0.2079	1.0651	−0.1860	0.077*	
C3	0.0504 (5)	1.0002 (3)	−0.1544 (3)	0.0630 (13)	
H3	−0.0062	1.0377	−0.1934	0.076*	
C4	0.0039 (5)	0.9259 (3)	−0.1000 (3)	0.0495 (11)	
C5	−0.1263 (5)	0.9048 (3)	−0.1012 (3)	0.0559 (11)	
H5	−0.1865	0.9408	−0.1390	0.067*	
C6	−0.1641 (5)	0.8330 (3)	−0.0479 (3)	0.0570 (12)	
H6	−0.2493	0.8194	−0.0504	0.068*	
C7	−0.0754 (4)	0.7777 (3)	0.0121 (3)	0.0486 (10)	
C8	−0.1120 (5)	0.7038 (3)	0.0705 (3)	0.0597 (12)	
H8	−0.1970	0.6883	0.0712	0.072*	
C9	−0.0219 (5)	0.6555 (3)	0.1258 (3)	0.0561 (12)	
H9	−0.0447	0.6057	0.1636	0.067*	
C10	0.1054 (4)	0.6791 (3)	0.1273 (3)	0.0439 (10)	
C11	0.0545 (4)	0.7974 (3)	0.0149 (3)	0.0416 (9)	
C12	0.0954 (4)	0.8733 (3)	−0.0432 (3)	0.0453 (10)	
C13	0.1957 (4)	0.6275 (3)	0.1945 (3)	0.0426 (10)	
C14	0.1613 (5)	0.6522 (3)	0.2786 (3)	0.0534 (11)	
C15	0.2371 (5)	0.6000 (4)	0.3422 (3)	0.0643 (13)	
H15	0.2143	0.6169	0.3986	0.077*	
C16	0.3449 (5)	0.5241 (3)	0.3256 (3)	0.0636 (13)	
C17	0.3784 (4)	0.5010 (3)	0.2401 (3)	0.0590 (12)	
H17	0.4514	0.4501	0.2274	0.071*	
C18	0.3066 (4)	0.5515 (3)	0.1736 (3)	0.0468 (10)	
C19	0.0407 (6)	0.7335 (4)	0.3020 (3)	0.0854 (17)	
H19A	0.0311	0.7392	0.3632	0.128*	
H19B	−0.0465	0.7250	0.2907	0.128*	
H19C	0.0612	0.7878	0.2670	0.128*	
C20	0.4251 (6)	0.4690 (4)	0.3969 (4)	0.101 (2)	
H20A	0.4882	0.5018	0.4085	0.152*	
H20B	0.4794	0.4118	0.3781	0.152*	
H20C	0.3588	0.4584	0.4495	0.152*	
C21	0.3423 (5)	0.5227 (3)	0.0829 (3)	0.0688 (14)	
H21A	0.4169	0.4679	0.0819	0.103*	
H21B	0.3734	0.5701	0.0411	0.103*	
H21C	0.2593	0.5119	0.0679	0.103*	
C22	0.9067 (6)	0.0232 (3)	0.3965 (3)	0.0731 (15)	
C23	1.0084 (9)	−0.0587 (4)	0.4057 (4)	0.094 (2)	
H23	0.9818	−0.1125	0.4306	0.113*	
C24	1.1494 (8)	−0.0579 (4)	0.3770 (4)	0.091 (2)	
H24	1.2194	−0.1118	0.3828	0.109*	
C25	1.1888 (6)	0.0243 (3)	0.3387 (3)	0.0717 (15)	
C26	1.3299 (6)	0.0325 (4)	0.3070 (4)	0.0821 (17)	
H26	1.4042	−0.0194	0.3092	0.099*	
C27	1.3598 (6)	0.1128 (4)	0.2739 (4)	0.0818 (17)	
H27	1.4544	0.1155	0.2547	0.098*	
C28	1.2495 (5)	0.1951 (3)	0.2671 (3)	0.0598 (12)	
C29	1.2745 (5)	0.2805 (4)	0.2325 (3)	0.0695 (14)	
H29	1.3666	0.2873	0.2107	0.083*	
C30	1.1608 (5)	0.3536 (3)	0.2314 (3)	0.0642 (13)	
H30	1.1763	0.4109	0.2087	0.077*	
C31	1.0218 (4)	0.3453 (3)	0.2633 (3)	0.0478 (10)	
C32	1.1082 (5)	0.1893 (3)	0.2975 (3)	0.0511 (11)	
C33	1.0767 (5)	0.1025 (3)	0.3340 (3)	0.0558 (12)	
C34	0.8998 (4)	0.4272 (3)	0.2636 (3)	0.0459 (10)	
C35	0.8679 (5)	0.4817 (3)	0.3307 (3)	0.0503 (11)	
C36	0.7599 (5)	0.5611 (3)	0.3259 (3)	0.0579 (12)	
H36	0.7384	0.5983	0.3701	0.069*	
C37	0.6836 (5)	0.5861 (3)	0.2567 (3)	0.0577 (12)	
C38	0.7163 (5)	0.5302 (3)	0.1918 (3)	0.0574 (12)	
H38	0.6648	0.5458	0.1457	0.069*	
C39	0.8230 (5)	0.4519 (3)	0.1937 (3)	0.0537 (11)	
C40	0.9502 (5)	0.4564 (3)	0.4063 (3)	0.0662 (13)	
H40A	0.9447	0.3965	0.4345	0.099*	
H40B	0.9102	0.4994	0.4479	0.099*	
H40C	1.0481	0.4573	0.3844	0.099*	
C41	0.5680 (5)	0.6738 (3)	0.2527 (4)	0.0894 (18)	
H41A	0.5216	0.6852	0.3113	0.134*	
H41B	0.4995	0.6684	0.2197	0.134*	
H41C	0.6098	0.7234	0.2245	0.134*	
C42	0.8605 (6)	0.3948 (4)	0.1182 (3)	0.0828 (16)	
H42A	0.7796	0.4059	0.0893	0.124*	
H42B	0.8866	0.3314	0.1407	0.124*	
H42C	0.9390	0.4110	0.0769	0.124*	
C43	0.6125 (7)	−0.2104 (6)	0.5048 (6)	0.152 (3)	
H43A	0.5746	−0.1630	0.5431	0.182*	
H43B	0.5526	−0.2534	0.5209	0.182*	

### Atomic displacement parameters (Å^2^)

**Table:**

	*U*^11^	*U*^22^	*U*^33^	*U*^12^	*U*^13^	*U*^23^
Co1	0.0409 (3)	0.0501 (3)	0.0512 (4)	−0.0109 (3)	−0.0104 (3)	−0.0055 (3)
Co2	0.0542 (4)	0.0565 (4)	0.0653 (4)	−0.0192 (3)	−0.0143 (3)	−0.0068 (3)
Cl1	0.0669 (8)	0.0820 (9)	0.0832 (9)	−0.0381 (7)	−0.0056 (7)	−0.0007 (7)
Cl2	0.0776 (8)	0.0572 (7)	0.0661 (8)	−0.0037 (6)	−0.0261 (7)	−0.0147 (6)
Cl3	0.0477 (7)	0.0725 (8)	0.0851 (9)	−0.0073 (6)	−0.0034 (6)	−0.0230 (7)
Cl4	0.1371 (15)	0.0841 (11)	0.1283 (15)	−0.0663 (10)	−0.0125 (12)	0.0002 (10)
Cl5	0.0840 (10)	0.1128 (12)	0.1225 (13)	−0.0340 (9)	−0.0530 (10)	−0.0117 (10)
Cl6	0.0909 (10)	0.0705 (8)	0.0697 (8)	−0.0218 (7)	−0.0053 (7)	−0.0116 (7)
Cl7	0.1138 (15)	0.187 (2)	0.1112 (15)	−0.0117 (14)	−0.0297 (12)	−0.0040 (14)
Cl8	0.218 (3)	0.149 (2)	0.278 (4)	−0.063 (2)	−0.165 (3)	0.049 (2)
N1	0.045 (2)	0.047 (2)	0.047 (2)	−0.0096 (16)	−0.0074 (16)	−0.0038 (17)
N2	0.0408 (18)	0.0408 (18)	0.0384 (18)	−0.0072 (15)	−0.0076 (15)	−0.0043 (15)
N3	0.086 (3)	0.045 (2)	0.056 (2)	−0.019 (2)	−0.016 (2)	−0.0078 (18)
N4	0.048 (2)	0.044 (2)	0.047 (2)	−0.0085 (17)	−0.0143 (16)	−0.0060 (16)
C1	0.060 (3)	0.056 (3)	0.051 (3)	−0.019 (2)	−0.002 (2)	0.001 (2)
C2	0.080 (4)	0.051 (3)	0.059 (3)	−0.019 (3)	−0.011 (3)	0.007 (2)
C3	0.073 (3)	0.056 (3)	0.059 (3)	−0.008 (3)	−0.026 (3)	0.003 (2)
C4	0.056 (3)	0.042 (2)	0.049 (3)	−0.008 (2)	−0.012 (2)	−0.002 (2)
C5	0.057 (3)	0.054 (3)	0.057 (3)	−0.004 (2)	−0.026 (2)	−0.003 (2)
C6	0.051 (3)	0.056 (3)	0.069 (3)	−0.008 (2)	−0.028 (2)	−0.006 (2)
C7	0.046 (2)	0.048 (2)	0.052 (3)	−0.011 (2)	−0.012 (2)	−0.003 (2)
C8	0.046 (3)	0.061 (3)	0.077 (3)	−0.020 (2)	−0.020 (2)	0.003 (3)
C9	0.052 (3)	0.054 (3)	0.065 (3)	−0.022 (2)	−0.019 (2)	0.010 (2)
C10	0.041 (2)	0.046 (2)	0.043 (2)	−0.0092 (19)	−0.0081 (19)	−0.0029 (19)
C11	0.036 (2)	0.044 (2)	0.044 (2)	−0.0037 (18)	−0.0097 (19)	−0.0082 (19)
C12	0.042 (2)	0.045 (2)	0.045 (2)	−0.0051 (19)	−0.007 (2)	−0.0073 (19)
C13	0.046 (2)	0.044 (2)	0.042 (2)	−0.0171 (19)	−0.0109 (19)	−0.0014 (19)
C14	0.058 (3)	0.057 (3)	0.046 (3)	−0.020 (2)	−0.003 (2)	−0.007 (2)
C15	0.076 (3)	0.081 (4)	0.041 (3)	−0.034 (3)	−0.008 (3)	−0.001 (3)
C16	0.070 (3)	0.069 (3)	0.058 (3)	−0.031 (3)	−0.028 (3)	0.015 (3)
C17	0.044 (3)	0.048 (3)	0.085 (4)	−0.009 (2)	−0.020 (3)	0.002 (2)
C18	0.041 (2)	0.046 (2)	0.052 (3)	−0.012 (2)	−0.006 (2)	−0.003 (2)
C19	0.099 (4)	0.077 (4)	0.071 (4)	−0.010 (3)	0.010 (3)	−0.030 (3)
C20	0.102 (5)	0.123 (5)	0.085 (4)	−0.042 (4)	−0.056 (4)	0.044 (4)
C21	0.079 (3)	0.061 (3)	0.060 (3)	−0.009 (3)	−0.002 (3)	−0.017 (2)
C22	0.107 (4)	0.055 (3)	0.065 (3)	−0.027 (3)	−0.019 (3)	−0.007 (3)
C23	0.163 (7)	0.044 (3)	0.075 (4)	−0.028 (4)	−0.019 (4)	−0.006 (3)
C24	0.144 (6)	0.048 (3)	0.072 (4)	0.006 (4)	−0.031 (4)	−0.012 (3)
C25	0.098 (4)	0.057 (3)	0.057 (3)	0.006 (3)	−0.031 (3)	−0.017 (2)
C26	0.075 (4)	0.078 (4)	0.086 (4)	0.021 (3)	−0.033 (3)	−0.025 (3)
C27	0.057 (3)	0.094 (4)	0.093 (4)	0.006 (3)	−0.021 (3)	−0.035 (4)
C28	0.050 (3)	0.068 (3)	0.059 (3)	0.003 (2)	−0.017 (2)	−0.020 (2)
C29	0.058 (3)	0.079 (4)	0.076 (4)	−0.024 (3)	−0.011 (3)	−0.011 (3)
C30	0.056 (3)	0.062 (3)	0.077 (4)	−0.022 (3)	−0.012 (3)	−0.002 (3)
C31	0.049 (3)	0.051 (3)	0.046 (2)	−0.011 (2)	−0.017 (2)	−0.005 (2)
C32	0.053 (3)	0.055 (3)	0.048 (3)	−0.010 (2)	−0.014 (2)	−0.011 (2)
C33	0.066 (3)	0.051 (3)	0.052 (3)	−0.003 (2)	−0.024 (2)	−0.011 (2)
C34	0.051 (2)	0.045 (2)	0.044 (2)	−0.016 (2)	−0.016 (2)	0.0045 (19)
C35	0.055 (3)	0.054 (3)	0.046 (3)	−0.019 (2)	−0.014 (2)	−0.002 (2)
C36	0.060 (3)	0.050 (3)	0.064 (3)	−0.017 (2)	−0.005 (3)	−0.010 (2)
C37	0.048 (3)	0.050 (3)	0.073 (3)	−0.015 (2)	−0.017 (2)	0.007 (2)
C38	0.050 (3)	0.055 (3)	0.071 (3)	−0.016 (2)	−0.026 (2)	0.007 (2)
C39	0.061 (3)	0.059 (3)	0.048 (3)	−0.023 (2)	−0.017 (2)	0.000 (2)
C40	0.075 (3)	0.073 (3)	0.059 (3)	−0.022 (3)	−0.027 (3)	−0.006 (3)
C41	0.064 (3)	0.060 (3)	0.134 (5)	−0.002 (3)	−0.022 (3)	0.002 (3)
C42	0.101 (4)	0.087 (4)	0.067 (3)	−0.013 (3)	−0.036 (3)	−0.013 (3)
C43	0.092 (5)	0.168 (8)	0.167 (8)	−0.005 (5)	−0.021 (5)	0.014 (7)

### Geometric parameters (Å, °)

**Table:**

Co1—N2	2.067 (3)	C19—H19A	0.9600
Co1—N1	2.090 (3)	C19—H19B	0.9600
Co1—Cl3	2.2130 (12)	C19—H19C	0.9600
Co1—Cl2	2.2146 (13)	C20—H20A	0.9600
Co2—N4	2.043 (3)	C20—H20B	0.9600
Co2—N3	2.071 (4)	C20—H20C	0.9600
Co2—Cl5	2.2028 (16)	C21—H21A	0.9600
Co2—Cl6	2.2092 (15)	C21—H21B	0.9600
Cl1—C1	1.719 (5)	C21—H21C	0.9600
Cl4—C22	1.719 (6)	C22—C23	1.391 (8)
Cl7—C43	1.656 (7)	C23—C24	1.376 (8)
Cl8—C43	1.717 (8)	C23—H23	0.9300
N1—C1	1.315 (5)	C24—C25	1.418 (8)
N1—C12	1.369 (5)	C24—H24	0.9300
N2—C10	1.329 (5)	C25—C33	1.403 (6)
N2—C11	1.375 (5)	C25—C26	1.412 (7)
N3—C22	1.319 (6)	C26—C27	1.336 (7)
N3—C33	1.371 (6)	C26—H26	0.9300
N4—C31	1.341 (5)	C27—C28	1.436 (7)
N4—C32	1.373 (5)	C27—H27	0.9300
C1—C2	1.402 (6)	C28—C29	1.395 (7)
C2—C3	1.359 (6)	C28—C32	1.401 (6)
C2—H2	0.9300	C29—C30	1.361 (7)
C3—C4	1.421 (6)	C29—H29	0.9300
C3—H3	0.9300	C30—C31	1.393 (6)
C4—C12	1.397 (6)	C30—H30	0.9300
C4—C5	1.412 (6)	C31—C34	1.492 (6)
C5—C6	1.350 (6)	C32—C33	1.443 (6)
C5—H5	0.9300	C34—C35	1.389 (6)
C6—C7	1.428 (6)	C34—C39	1.400 (6)
C6—H6	0.9300	C35—C36	1.392 (6)
C7—C11	1.405 (5)	C35—C40	1.510 (6)
C7—C8	1.406 (6)	C36—C37	1.388 (6)
C8—C9	1.355 (6)	C36—H36	0.9300
C8—H8	0.9300	C37—C38	1.376 (6)
C9—C10	1.404 (6)	C37—C41	1.517 (6)
C9—H9	0.9300	C38—C39	1.372 (6)
C10—C13	1.500 (5)	C38—H38	0.9300
C11—C12	1.442 (5)	C39—C42	1.520 (6)
C13—C14	1.384 (6)	C40—H40A	0.9600
C13—C18	1.405 (5)	C40—H40B	0.9600
C14—C15	1.380 (6)	C40—H40C	0.9600
C14—C19	1.516 (6)	C41—H41A	0.9600
C15—C16	1.375 (7)	C41—H41B	0.9600
C15—H15	0.9300	C41—H41C	0.9600
C16—C17	1.396 (6)	C42—H42A	0.9600
C16—C20	1.505 (7)	C42—H42B	0.9600
C17—C18	1.386 (6)	C42—H42C	0.9600
C17—H17	0.9300	C43—H43A	0.9700
C18—C21	1.504 (6)	C43—H43B	0.9700
			
N2—Co1—N1	81.74 (13)	C16—C20—H20C	109.5
N2—Co1—Cl3	117.08 (9)	H20A—C20—H20C	109.5
N1—Co1—Cl3	110.81 (10)	H20B—C20—H20C	109.5
N2—Co1—Cl2	111.88 (9)	C18—C21—H21A	109.5
N1—Co1—Cl2	109.88 (10)	C18—C21—H21B	109.5
Cl3—Co1—Cl2	119.14 (5)	H21A—C21—H21B	109.5
N4—Co2—N3	81.41 (15)	C18—C21—H21C	109.5
N4—Co2—Cl5	117.29 (11)	H21A—C21—H21C	109.5
N3—Co2—Cl5	108.36 (12)	H21B—C21—H21C	109.5
N4—Co2—Cl6	111.17 (10)	N3—C22—C23	124.5 (6)
N3—Co2—Cl6	107.10 (11)	N3—C22—Cl4	116.2 (4)
Cl5—Co2—Cl6	122.82 (7)	C23—C22—Cl4	119.3 (5)
C1—N1—C12	117.8 (4)	C24—C23—C22	118.1 (5)
C1—N1—Co1	130.8 (3)	C24—C23—H23	120.9
C12—N1—Co1	111.4 (3)	C22—C23—H23	120.9
C10—N2—C11	118.7 (3)	C23—C24—C25	120.5 (5)
C10—N2—Co1	129.9 (3)	C23—C24—H24	119.7
C11—N2—Co1	111.3 (2)	C25—C24—H24	119.7
C22—N3—C33	117.0 (4)	C33—C25—C26	119.0 (5)
C22—N3—Co2	130.8 (4)	C33—C25—C24	115.9 (6)
C33—N3—Co2	112.0 (3)	C26—C25—C24	125.0 (5)
C31—N4—C32	117.9 (4)	C27—C26—C25	121.9 (5)
C31—N4—Co2	129.3 (3)	C27—C26—H26	119.1
C32—N4—Co2	112.8 (3)	C25—C26—H26	119.1
N1—C1—C2	123.2 (4)	C26—C27—C28	121.6 (5)
N1—C1—Cl1	117.5 (4)	C26—C27—H27	119.2
C2—C1—Cl1	119.2 (4)	C28—C27—H27	119.2
C3—C2—C1	118.8 (4)	C29—C28—C32	118.0 (4)
C3—C2—H2	120.6	C29—C28—C27	124.0 (5)
C1—C2—H2	120.6	C32—C28—C27	118.0 (5)
C2—C3—C4	120.6 (4)	C30—C29—C28	118.3 (5)
C2—C3—H3	119.7	C30—C29—H29	120.8
C4—C3—H3	119.7	C28—C29—H29	120.8
C12—C4—C5	120.7 (4)	C29—C30—C31	122.0 (5)
C12—C4—C3	115.8 (4)	C29—C30—H30	119.0
C5—C4—C3	123.5 (4)	C31—C30—H30	119.0
C6—C5—C4	120.5 (4)	N4—C31—C30	120.9 (4)
C6—C5—H5	119.7	N4—C31—C34	118.4 (4)
C4—C5—H5	119.7	C30—C31—C34	120.7 (4)
C5—C6—C7	121.0 (4)	N4—C32—C28	122.9 (4)
C5—C6—H6	119.5	N4—C32—C33	116.9 (4)
C7—C6—H6	119.5	C28—C32—C33	120.2 (4)
C11—C7—C8	117.4 (4)	N3—C33—C25	123.9 (5)
C11—C7—C6	119.6 (4)	N3—C33—C32	116.8 (4)
C8—C7—C6	123.1 (4)	C25—C33—C32	119.3 (5)
C9—C8—C7	119.1 (4)	C35—C34—C39	120.1 (4)
C9—C8—H8	120.4	C35—C34—C31	119.9 (4)
C7—C8—H8	120.4	C39—C34—C31	119.9 (4)
C8—C9—C10	121.3 (4)	C34—C35—C36	118.5 (4)
C8—C9—H9	119.3	C34—C35—C40	120.9 (4)
C10—C9—H9	119.3	C36—C35—C40	120.7 (4)
N2—C10—C9	120.9 (4)	C37—C36—C35	121.8 (4)
N2—C10—C13	120.1 (3)	C37—C36—H36	119.1
C9—C10—C13	119.0 (4)	C35—C36—H36	119.1
N2—C11—C7	122.5 (4)	C38—C37—C36	118.5 (4)
N2—C11—C12	118.3 (3)	C38—C37—C41	121.1 (5)
C7—C11—C12	119.1 (4)	C36—C37—C41	120.4 (5)
N1—C12—C4	123.8 (4)	C39—C38—C37	121.5 (4)
N1—C12—C11	117.2 (4)	C39—C38—H38	119.2
C4—C12—C11	119.0 (4)	C37—C38—H38	119.2
C14—C13—C18	120.9 (4)	C38—C39—C34	119.6 (4)
C14—C13—C10	118.8 (4)	C38—C39—C42	119.7 (4)
C18—C13—C10	120.1 (4)	C34—C39—C42	120.6 (4)
C15—C14—C13	118.7 (4)	C35—C40—H40A	109.5
C15—C14—C19	120.0 (4)	C35—C40—H40B	109.5
C13—C14—C19	121.3 (4)	H40A—C40—H40B	109.5
C16—C15—C14	122.8 (4)	C35—C40—H40C	109.5
C16—C15—H15	118.6	H40A—C40—H40C	109.5
C14—C15—H15	118.6	H40B—C40—H40C	109.5
C15—C16—C17	117.5 (4)	C37—C41—H41A	109.5
C15—C16—C20	121.1 (5)	C37—C41—H41B	109.5
C17—C16—C20	121.4 (5)	H41A—C41—H41B	109.5
C18—C17—C16	122.1 (4)	C37—C41—H41C	109.5
C18—C17—H17	118.9	H41A—C41—H41C	109.5
C16—C17—H17	118.9	H41B—C41—H41C	109.5
C17—C18—C13	118.0 (4)	C39—C42—H42A	109.5
C17—C18—C21	120.7 (4)	C39—C42—H42B	109.5
C13—C18—C21	121.3 (4)	H42A—C42—H42B	109.5
C14—C19—H19A	109.5	C39—C42—H42C	109.5
C14—C19—H19B	109.5	H42A—C42—H42C	109.5
H19A—C19—H19B	109.5	H42B—C42—H42C	109.5
C14—C19—H19C	109.5	Cl7—C43—Cl8	113.2 (5)
H19A—C19—H19C	109.5	Cl7—C43—H43A	108.9
H19B—C19—H19C	109.5	Cl8—C43—H43A	108.9
C16—C20—H20A	109.5	Cl7—C43—H43B	108.9
C16—C20—H20B	109.5	Cl8—C43—H43B	108.9
H20A—C20—H20B	109.5	H43A—C43—H43B	107.8

### Hydrogen-bond geometry (Å, °)

**Table:**

*D*—H···*A*	*D*—H	H···*A*	*D*···*A*	*D*—H···*A*
C9—H9···Cg1^i^	0.93	2.59	3.470 (5)	157

Symmetry codes: (i) *x*−1, *y*, *z*.

### Table 2 Possible π–π stacking interactions (Å, °)

**Table:**

	Centroid–centroid	α	CgI-perp	CgJ-perp	slippage
Cg2 – Cg2^i^	3.877 (3)	0.0	3.712	3.712	1.12
Cg2 – Cg3^i^	3.923 (3)	0.49	3.712	3.701	1.23
Cg4 – Cg4^ii^	3.992 (3)	0.02	3.490	3.490	1.94

Symmetry codes: (i) -x, 2-y, -z; (ii) 2-x, -y, 1-z. CgI -CgJ = distance between ring Centroids; α = dihedral angle between Planes I and J; CgI–perp = perpendicular distance of Cg(I) from ring J; CgJ–perp = perpendicular distance of Cg(J) from ring I; slippage = distance between Cg(I) and perpendicular projection of Cg(J) on Ring I. Cg2 is the centroid of atoms N1,C1–C4,C12; Cg3 is the centroid of atoms C4–C7,C11,C12; Cg4 is the centroid of atoms N3,C22–C25,C33.
